# Natural scenes reveal diverse representations of 2D and 3D body pose in the human brain

**DOI:** 10.1073/pnas.2317707121

**Published:** 2024-06-03

**Authors:** Hongru Zhu, Yijun Ge, Alexander Bratch, Alan Yuille, Kendrick Kay, Daniel Kersten

**Affiliations:** ^a^Department of Cognitive Science, Johns Hopkins University, Baltimore, MD 21218; ^b^Department of Psychology, University of Minnesota, Minneapolis, MN 55455; ^c^Laboratory for Consciousness, Riken Center for Brain Science, Wako, Saitama 3510198, Japan; ^d^Center for Magnetic Resonance Research, Department of Radiology, University of Minnesota, Minneapolis, MN 55455

**Keywords:** visual cortex, body perception, 3D pose, natural images

## Abstract

Human pose, defined as the spatial relationships between body parts, carries instrumental information supporting the understanding of motion and action of a person. A substantial body of previous work has identified cortical areas responsive to images of bodies and different body parts. However, the neural basis underlying the visual perception of body part relationships has received less attention. To broaden our understanding of body perception, we analyzed high-resolution fMRI responses to a wide range of poses from over 4,000 complex natural scenes. Using ground-truth annotations and an application of three-dimensional (3D) pose reconstruction algorithms, we compared similarity patterns of cortical activity with similarity patterns built from human pose models with different levels of depth availability and viewpoint dependency. Targeting the challenge of explaining variance in complex natural image responses with interpretable models, we achieved statistically significant correlations between pose models and cortical activity patterns (though performance levels are substantially lower than the noise ceiling). We found that the 3D view-independent pose model, compared with two-dimensional models, better captures the activation from distinct cortical areas, including the right posterior superior temporal sulcus (pSTS). These areas, together with other pose-selective regions in the LOTC, form a broader, distributed cortical network with greater view-tolerance in more anterior patches. We interpret these findings in light of the computational complexity of natural body images, the wide range of visual tasks supported by pose structures, and possible shared principles for view-invariant processing between articulated objects and ordinary, rigid objects.

## Significance

The visual processing of human bodies is important for social and cognitive functions. While previous studies have identified brain regions involved in detecting bodies and body parts, understanding how the brain processes three-dimensional (3D) spatial arrangements of body parts seen in everyday life has remained a challenge. To address this challenge, we used 3D reconstruction algorithms to extract 3D body pose from a large set of natural images and analyzed human brain responses to these images. We found a distributed cortical network encoding body pose, with two-dimensional pose better represented in the lateral-occipital-temporal cortex (LOTC) and 3D pose in both LOTC and posterior superior temporal sulcus (pSTS). We highlight the importance of considering different pose representations for different visual tasks.

As highly social creatures, our visual world is filled with images of the bodies of other humans. The perception of human bodies provides crucial support for understanding people’s actions, intentions, and social interactions. Despite the dynamic nature of bodies, human vision is able to make accurate inferences from even a single image. How does the human visual system support its abilities to interpret the actions of others? Part of the answer requires understanding how the brain computes and represents the spatial relationships between body parts, defined here as pose. Estimating human pose from a natural image is a challenging computational problem due to both the complexity of natural images (1, 2) and the wide range of behavioral tasks.

The complexity of natural images is the result of an enormous range of variations in viewpoint, lighting, occlusion, and background clutter. The computational problem for bodies, in contrast to faces for example, is especially compounded by clothing variations and the large space of joint articulations resulting in self-occlusions. Critically, computing pose requires segmenting image regions with body parts from those of other parts and the background clutter. Past studies using functional magnetic imaging (fMRI) in humans have discovered several cortical areas likely involved in the solutions to these problems (3, 4). However, past studies have only measured responses to highly simplified stimuli in which the image of the body is easily segmented and over a very restricted range of poses (3–11). Our understanding of the neural basis of human pose perception has thus far been limited by the lack of natural image datasets representative of the enormous pose space of everyday visual experience but also by the lack of annotations that parameterize body poses.

The interpretation of images of bodies requires different kinds of pose parameterizations depending on the behavioral task. Some tasks can be performed with only knowledge of two-dimensional (2D) relationships between parts, but others require knowledge of three-dimensional (3D) depth relationships. Research in computer vision has shown that computing 2D pose can be done to a limited extent using image-based methods that rely on identifying parts and spatial relationships within the image (1, 12). However, computing 3D pose requires prior domain-specific knowledge of human 3D body structure (13, 14). Further, other tasks require estimation of body orientation relative to a direction of interest such as with respect to the viewer, other objects, or people. Understanding how cortical activity depends on the perceptual task requires an efficient way of extracting 2D, 3D, and view annotations.

To address the need for a large range of natural image variations, we exploited the high-quality measurements of brain responses to complex natural scenes available in the Natural Scenes Dataset (NSD) (15). The NSD is a massive high-resolution 7T fMRI dataset containing responses to over 70,000 natural scenes taken from the Microsoft Common Objects in Context (COCO) database (16). The data were collected on eight subjects in a continuous recognition task where subjects were instructed to indicate whether they have seen each presented image at any point in the current or past sessions. The NSD memory task taps into different types and levels of processing while avoiding introducing attentional demands related to object-specific recognition tasks, including body decisions. We identified a subset of 4,450 natural images that contain single persons engaged in various activities and thus spanning a large range of poses, appearances, and backgrounds.

To address the need for distinct pose parameterizations, we used the 2D annotations from the COCO image dataset. However, the COCO dataset does not supply the depths of the joints or the body viewpoints. We used a 3D human mesh reconstruction algorithm (17) to compute the 3D body pose and overall body orientation for each image. We then tested different models of body pose representation using the approach of representational similarity analysis (RSA) and searchlight mapping (18, 19).

Our study addresses three main questions. First, the complexity of natural image computations required to estimate pose for a range of tasks raises the question of body-specific responses in a wider range of cortical areas, including early visual areas, than previous studies have found. Second, different task requirements suggest the possibility of visual areas differing in their sensitivity to relative depth in poses, for example, areas that distinguish between view-dependent 2D and view-dependent 3D pose information. Likewise, given the utility of view-independent representations, there might also exist areas sensitive to view-independent 3D pose information. Third, to be able to relate our results to previous ROI-based studies of body sensitivity, we compared pose activations to several control models, specifically testing for cortical regions sensitive to body size and position (20, 21). We discuss how our results may relate to the possibility of shared mechanisms between bodies and other objects (22). We also discuss how our findings relate to the view-invariant processing for articulated objects, faces, and general objects. We study cortical responses to a large number of natural images of people over a wide range of poses, typical of normal activities, and show how these responses depend on viewpoint and 3D pose information.

## Results

We constructed four key pose models representing view-independent 3D pose, view-dependent 3D pose, view-dependent 2D pose, and the overall body orientation relative to viewpoint. The information in each model was characterized by a representational dissimilarity matrix (RDM) whose entries contained the distances between pose parameters in all pairs of images (18). The RDMs encapsulate which distinctions between stimuli are emphasized and which are de-emphasized for a given pose representation. The models were evaluated using RSA in which the correlations between model and neural RDMs were measured. The neural RDMs were constructed using searchlight mapping, in which a local window is slid across cortical voxels and dissimilarities of the responses of all voxels in the window are quantified for all image pairs. In addition, to comparing our results with previously studied cortical regions, we conducted ROI-based RSA with the same pose and viewpoint models, investigating neural correlations in early visual areas (V1, V2, V3), and established body (EBA, FBA) and face-sensitive areas (OFA and FFA).

### Pose Model and RDM Construction.

To compute different pose and viewpoint parameterizations for natural pose images, we used a 3D human mesh reconstruction algorithm (17) to extract human 3D body meshes that were further processed for the four pose models. These reconstructed human body meshes predicted fairly accurate and detailed pose information consistent with the poses in the complex natural images. *SI Appendix*, Fig. S1 shows examples of natural pose images used in the Natural Scenes Dataset together with their reconstructed meshes. These meshes were parameterized by 3D joint rotation angles for major joints excluding the fingers, toes, and jaw, as well as body global rotation angles that allow us to parameterize complex natural poses.

**Fig. 1.**
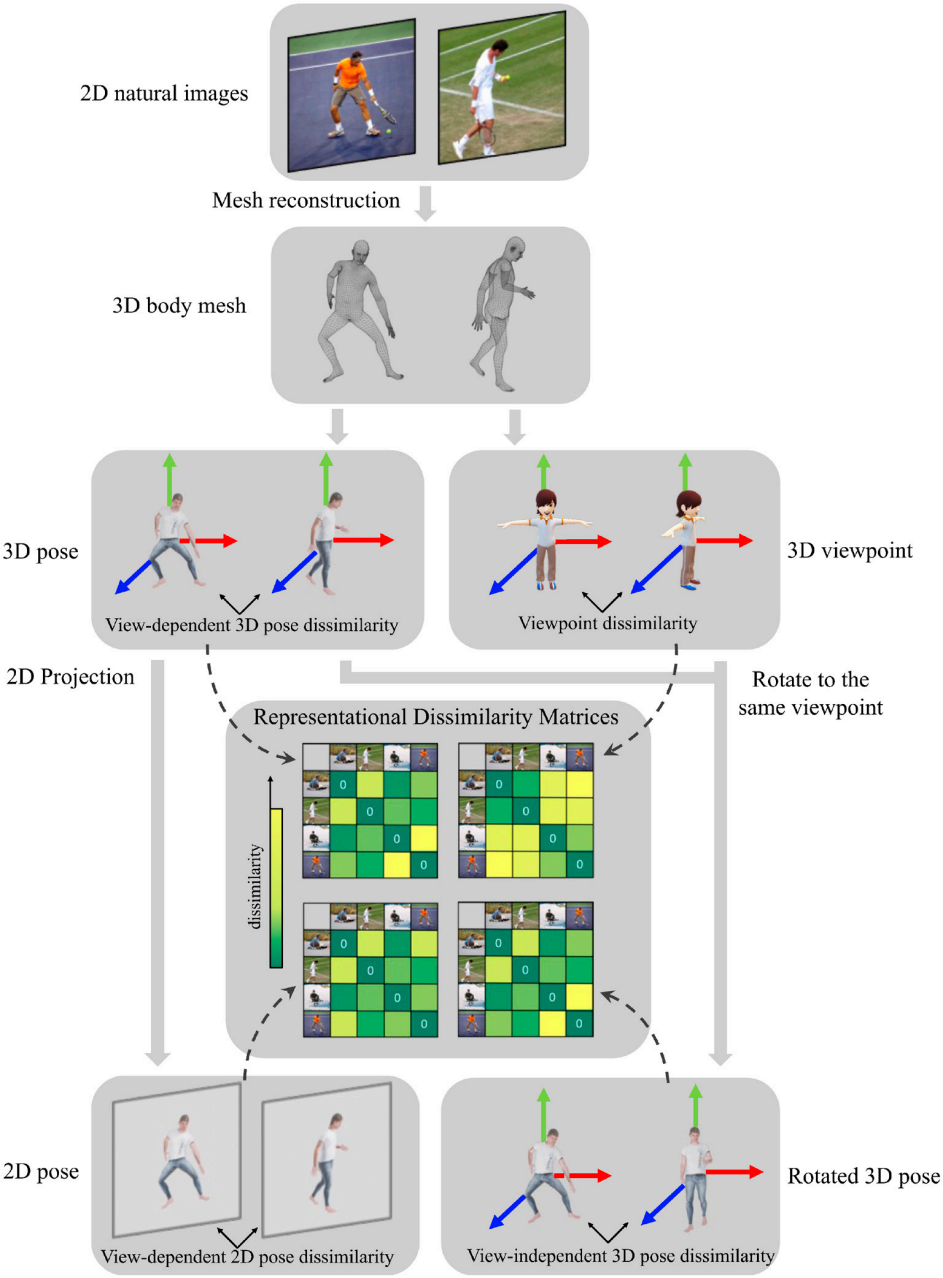
Illustration of our pose and viewpoint model RDM construction pipeline with an example pair of natural images. Cropped 2D natural images (first row) were taken as input to the pipeline. First, we reconstructed 3D body meshes from natural image input (second row). From these 3D mesh, we extracted 3D joint locations in the viewers’ frame of reference, which were used to calculate view-dependent 3D pose dissimilarities between image pairs. With the global body rotation parameter, we represented viewpoints as 3D rotations and characterized viewpoint dissimilarity with distances between two 3D rotation quaternions. By projecting joint locations from 3D to 2D and thus discarding depth information, we further calculated view-dependent 2D pose dissimilarity using 2D joint locations. As we got 3D viewpoint information, we rotated 3D poses to the same viewpoint (for example, to the same frontal view shown in this figure), and then computed the view-independent 3D pose dissimilarity using 3D joint positions from rotated human body meshes. These pairwise dissimilarity values are used as matrix entries to build different pose and viewpoint model RDMs.

The Fig. 1 shows the RDM building pipeline for pose and viewpoint models using reconstructed meshes. The body mesh algorithm returns 3D joint rotation and 3D body global rotation parameters, the latter containing the viewpoint information. We used each reconstructed 3D mesh to compute 3D joint positions and body orientation, namely the approximate direction the body is facing. All of the information was subsequently used to measure dissimilarities between images in our dataset. And these pairwise dissimilarities were organized into different RDMs used for RSA.

Annotations and 3D reconstructions were used to build the RDMs for the 4,450 natural images: a) view-independent 3D pose RDM, b) view-dependent 3D pose RDM, c) view-dependent 2D pose RDM, and d) viewpoint RDM. We visualized these RDMs by ordering 4,450 natural pose images according to their viewpoint and plotted the viewpoint distribution in *SI Appendix*, Fig. S2. Our view-independent pose RDM was intended to capture intrinsic, view-independent pose information, and it shows distinct patterns from other RDMs as well as lower correlation with the viewpoint RDM compared with other view-dependent pose RDMs.

### Pose Model RSA.

To identify different pose representations across visual cortical areas, we performed classic searchlight-based RSA with each of the four RDMs: a) view-independent 3D pose RDM, b) view-dependent 3D pose RDM, c) viewpoint-dependent 2D pose RDM, and d) viewpoint RDM. The reasons to favor classic over feature reweighted RSA are twofold. First, feature reweighting could lead completely different models to yield very similar brain predictivity by effectively solving for a similar subspace present in all the learned feature spaces (23). Classic RSA avoids this issue by the highly constrained linking assumption that all dimensions in a given model representation contribute equally to the population-level geometry. Second, in our specific case comparing 2D vs. 3D and view-dependent vs. view-independent poses, feature reweighting could diminish meaningful differences across pose models because 3D rotation and 2D projection are both linear transformations. In general, since there are not yet well-established principles in the field that favor one approach over the other, we opted for classic RSA, as it aligns better with our use case and addresses the aforementioned concerns.

### A Distributed Cortical Network Sensitive to the Geometric Structure of Body Pose.

**Fig. 2.**
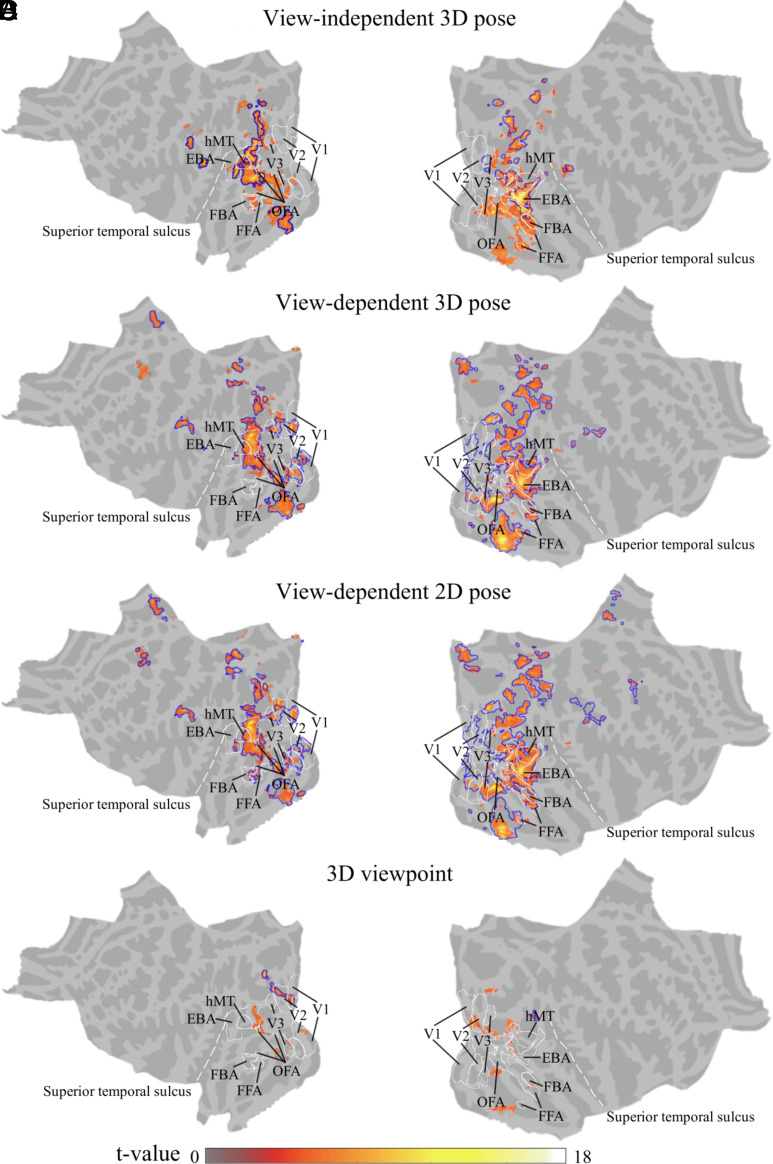
Group-level searchlight-based standard RSA results for the (*A*) view-independent 3D pose, (*B*) view-dependent 3D pose, (*C*) view-dependent 2D pose, and (*D*) 3D viewpoint model. Color maps show t-values for significant clusters that passed a cluster-based nonparametric analysis with Monte Carlo permutation (cluster stat: max sum; initial *P* < 0.001) from standard RSA. The correlation maps for each participant were first Fisher transformed to normal distribution and then the t scores were measured. ROI annotations were obtained from the functional atlas in ref. 24. All pose models showed clusters in the LOTC. View-independent 3D pose results also showed unique clusters along the right posterior STS (marked by dashed white lines). Blue contours denote clusters that are still statistically significant after performing partial correlation controls for low-level image and body size and location features. Inflated surface map of the results is shown in *SI Appendix*, Fig. S4*A*.

We present group-level whole-brain searchlight RSA results in Fig. 2, showing t-values within clusters that passed a cluster-based Monte Carlo permutation (cluster stat: max sum; initial *P* < 0.001). These results are compared against the functional region of interest (ROI) atlas from ref. 24. The lateral occipital-temporal cortex (LOTC) was defined as the area bounded by the middle portion of the middle temporal gyrus (MTG), the lateral occipital sulcus (LOS), the superior temporal sulcus (STS), and the inferior temporal gyrus (ITG) (25). For the view-independent 3D pose model (Fig. 2*A*), we found distributed clusters across the LOTC, fusiform gyrus, parahippocampal gyrus, posterior parietal cortex, as well as the temporal-parietal junction, including posterior superior temporal sulcus (pSTS). For the view-dependent 3D pose model (Fig. 2*B*), we also found a distributed pattern with major activated areas in the LOTC, fusiform gyrus, parahippocampal gyrus, and posterior parietal cortex. However, we found little or no significant clusters around pSTS. For the view-dependent 2D pose model (Fig. 2*C*), we found major activity patterns in cortical areas near the LOTC, fusiform gyrus, parahippocampal gyrus, and posterior parietal cortex. These results revealed a pattern of pose-sensitive voxels beyond body and face-related ROIs from past literature. In comparison of the positions of revealed pose-sensitive voxels with body and face ROIs, we found that pose activations from both view-dependent and view-independent pose models extended into parts of EBA and FFA. The 3D view-independent pose model showed stronger activations in FBA compared with other view-dependent pose models. With respect to body-sensitive responses in early visual areas, we found significant clusters near V1, V2, and V3, with a few extending into the posterior parietal cortex from both the 3D viewpoint model and other pose models.

Fig. 2 also shows results of additional analyses in which we rule out confounding factors of low-level image features, represented by HMAX C1 (26), and body-related control models for body size, body polar angle, and body polar distance, from each target pose model. The three body-related controls used measures of the angle and the distance of the body with respect to fixation, as well as the area of the body relative to the full image. Analyses were carried out within a partial correlation searchlight-based RSA framework. To facilitate comparison with standard RSA results, in Fig. 2 we overlay blue contours denoting clusters passing permutation tests with partial correlation controls. Notably, we observed similarly distributed pose clusters spanning the LOTC and fusiform gyrus, as well as smaller but still significant 3D view-independent pose clusters near pSTS and 2D/3D view-dependent pose clusters in V1. These analyses, together with the low correlations between pose model and control model RDMs (*SI Appendix*, Fig. S3), establish that our pose models do capture cortical sensitivity to poses rather than merely low-level features or activity due to the size and location of the body in the images. Consequently, our next set of analyses focused on cortical sensitivities to different aspects of pose information with comparisons among the four pose models.

### Cortical Sensitivity to Relative Body Depth and Intrinsic 3D Pose Information.

To identify visual areas differing in the sensitivity to relative depth in poses, we compared results from the view-dependent 3D pose and view-dependent 2D pose models. As the 3D pose model naturally has knowledge about 2D geometry, it is impossible to determine what aspects of pose information drove the RSA effects in the pose-sensitive voxels in standard RSA. Hence, we conducted a partial-correlation RSA using the view-dependent 3D pose RDM while regressing out the view-dependent 2D pose RDM. This measured the strength of relationships between the auxiliary depth information and the neural data by controlling for the effect from 2D poses. *SI Appendix*, Fig. S5 shows the group-level results as well as individual partial correlation maps from each subject. None of the clusters from the group-level results passed the cluster-based permutation test (cluster stat: max sum; init *P* < 0.001), which might be partly attributed to the high correlation (0.97) between our 2D and 3D view-dependent pose RDM. We found that seven out of eight subjects showed sensitivity to depth information in the LOTC after removing dependency on the 2D pose.

**Fig. 3.**
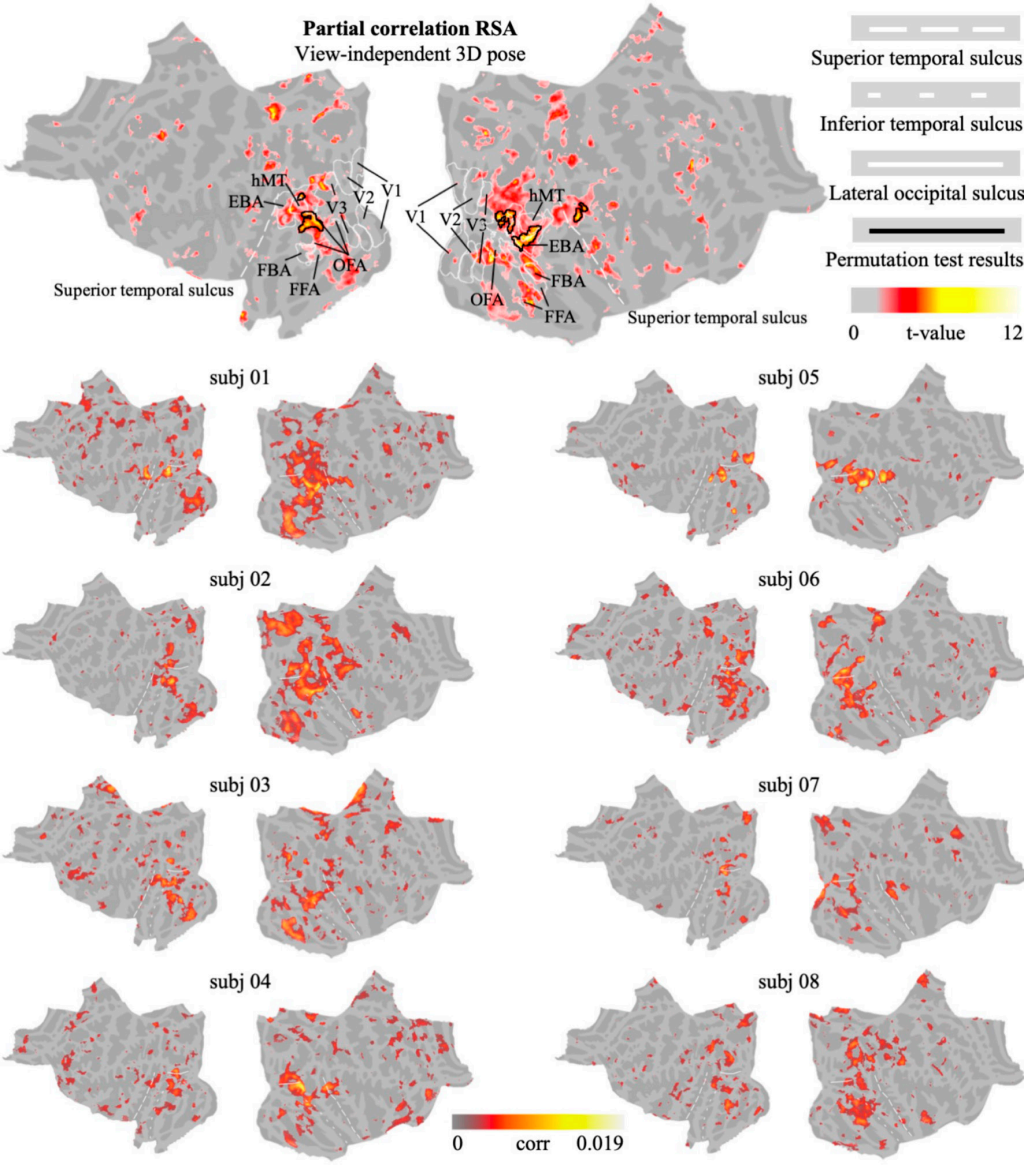
Group-level results (*Top*) as well as individual partial correlation maps highlighting areas responsive to 3D intrinsic, view-independent pose information, obtained after regressing out 2D and 3D view-dependent as well as viewpoint components from the 3D view-independent pose model. Color maps show t-values and the partial correlation values respectively for group-level and individual plots. Black contours outline the clusters that passed the cluster-based permutation test (cluster stat: max sum; init *P* < 0.001). These clusters are located in the LOTC as well as right posterior STS, and they show sensitivity to intrinsic 3D pose information after removing view-dependent components. Inflated surface versions of the individual results are included in *SI Appendix*, Fig. S7. Inflated surface maps of the group-level results are included in *SI Appendix*, Fig. S4*B*.

Similarly, to identify visual areas sensitive to intrinsic, view-independent pose information and to control for view-dependent components, we conducted another partial-correlation RSA using the view-independent 3D pose model while regressing out the effects from all the other view-dependent pose and viewpoint models. Fig. 3 shows group-level results and individual partial correlation maps. Again, we refined the group-level results using the cluster-based permutation test (cluster stat: max sum; init *P* < 0.001). Consistent with the results from group-level standard searchlight RSA, we found clusters sensitive to intrinsic 3D pose information in the LOTC as well as the right pSTS, after removing view-dependent components. These areas may encode intrinsic 3D pose information that is independent of the specific viewpoint.

### Relationship to Nearby ROIs.

**Fig. 4.**
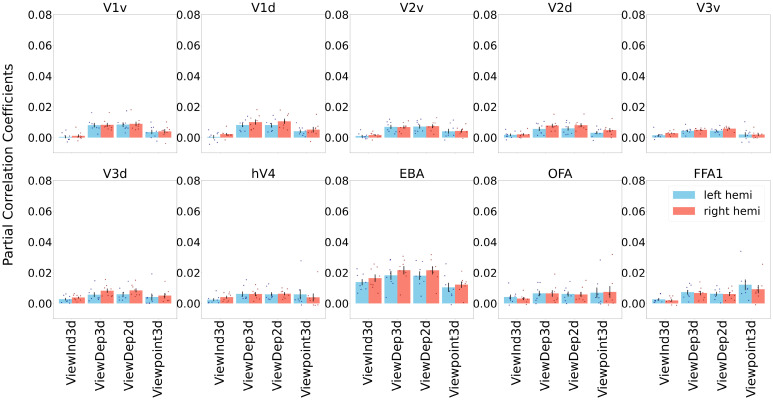
ROI-based RSA results for our pose models, obtained after regressing out low-level image and body features using partial correlations. Ten ROIs are selected including dorsal/ventral V1, dorsal/ventral V2, dorsal/ventral V3, V4, EBA, OFA, and FFA1. Blue and red colors denote ROIs from left and right hemispheres, respectively. The error bar shows the mean and the SE for the correlation between neural RDMs and model RDMs across eight subjects within each ROI. Each dot represents the correlation value from each individual subject., see *Materials and Methods* for the details of the ROI-based RSA procedure.

**Fig. 5.**
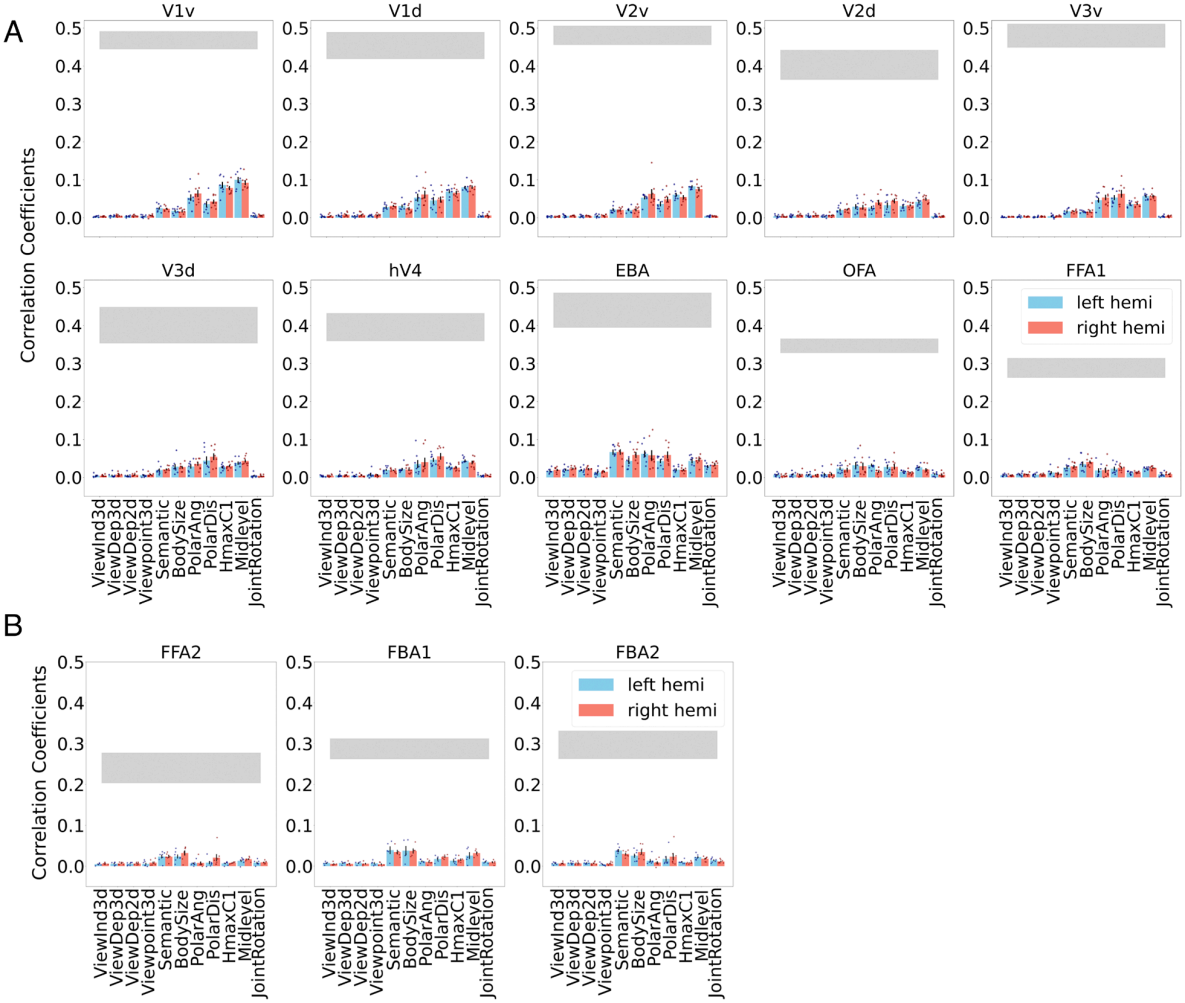
Standard ROI-based RSA results and noise ceiling measurements for pose model and control models. (*A*) Ten ROIs are selected with functional localizers, including dorsal/ventral V1, dorsal/ventral V2, dorsal/ventral V3, V4, EBA, OFA, and FFA1. These ROIs present in both hemispheres in all eight subjects. Blue and red colors denote ROIs from left and right hemispheres, respectively. Error bar plots show the mean and the SE for the correlation between neural RDMs and model RDMs across eight subjects within each ROI. Each dot represents the correlation value from each individual subject. The gray horizontal bar denotes noise ceiling, specifically, the mean noise ceiling and SE across subjects. (*B*) We separately present the results in FFA2, FBA1, and FBA2 because only a subset of subjects have FFA2 (n = 7), FBA1 (n = 5), and FBA2 (n = 7) in both hemispheres.

Many past studies of body perception adopted ROI-based analysis. To further validate and connect our searchlight RSA to these literatures, we conducted classic RSA on ROIs derived from functional localizers (15) after controlling the effects of HMAX C1 (26), body size, body polar angle, and body polar distance. Given the positional overlap of body ROIs and pose-sensitive voxels found in searchlight RSA, we expected that ROI-based RSA would also reveal stronger correlations for pose models in these areas. As shown in Fig. 4, ROI-based results are consistent with our searchlight results. Specifically, neural correlations of pose models are higher in EBA than in other ROIs. To obtain a better sense of overall model performance, we compared standard ROI RSA results for all pose and control models against the noise ceiling, i.e., the maximum performance that can be achieved given the level of noise in the data (Fig. 5). We observed relatively modest correlation values compared to the noise ceiling, a general observation that is commonly seen in classic RSA (23). We leave it for future work to get improved performance with techniques like voxel-wise fitting and further fine-tuning the pose models (e.g., incorporating low-level factors like body size).

### Control Model RSA.

In a final set of analyses, we conducted standard searchlight-based RSA on seven models, including HMAX C1, body polar angle, distance, and size control models used in the earlier partial correlation controls (*SI Appendix*, Fig. S8 *A–D*), together with three additional models to control for mid- and high-level features (*SI Appendix*, Fig. S8 *E–G*).

First, to capture high-level semantic-related activity, we designed a control based on image captions. A second additional control was designed to assess whether activity patterns depend on how the 3D view-independent pose model is parameterized. The original alignment-based view-independent 3D pose model captures overall pose structure using spatial distances between joints after alignment. In this model, a subtle change in the shoulder joint angle may lead to subsequent adjustments in the positions of the elbow and wrist. To allow for this, we constructed a joint rotation–based model based on differences in joint angles. While the joint rotation–based model might overlook these nuances, the original alignment model better captures them by considering the overall spatial relationships and configurations of the joints. Despite the model differences, we expect similar patterns in both models if clusters identified for the view-independent 3D pose model indeed encode information about view-independent poses.

A third additional model was constructed to ensure that our pose models did not merely capture mid-level features. In contrast to models trained for end-to-end object recognition, current body pose computer vision models rely on localization of multiple parts, suggesting the importance of early to mid-level features supporting localization and segmentation. As a measure of the contribution of mid-level features supporting pose, we used feature embeddings from the image encoder layer in segment anything model (SAM) (27). SAM is a computer vision foundation model trained on over 1 billion natural images to produce state-of-the-art accurate segmentations useful for a range of visual tasks.

The *SI Appendix*, Fig. S8 shows resulting clusters for control models from standard searchlight-based RSA in comparison with results from the original view-independent 3D pose model. Three body-related models produced significant clusters near the LOTC and fusiform gyrus, but none of them largely overlapped with the pose model. This is different from ROI results showing higher correlations for body controls in EBA and FBA (Fig. 5), possibly due to the inherent difference between ROI and searchlight approaches. ROI RSA is performed on ROIs in each subject’s individual volume space whereas searchlight RSA enforces consistent cross-subject anatomical positions in the MNI space in permutation tests. Parts of EBA and FBA may have higher correlation with body controls, but different subjects may not perfectly align when mapped to the MNI space. The HMAX C1 model produced clusters not only in the early visual cortex but also along the LOTC and the fusiform gyrus, including several high-level regions (e.g., regions involved in scene perception) that were shown to be sensitive to low-level features such as line orientation and textures (28–31). We found no largely overlapping clusters between the pose model and the HMAX C1 model. For the semantic control model, we found activation patterns in the LOTC, fusiform gyrus, parahippocampal gyrus, and posterior parietal cortex. Considering that the semantic model was built using image captions, the model is expected to encode some aspects of pose information, along with other information about objects, scenes and actions, etc. For the joint rotation–based view-independent control RDM, we found largely overlapping clusters across different cortical areas, including the LOTC, fusiform gyrus, posterior parietal cortex, as well as pSTS. For the mid-level feature model based on SAM embeddings, we found activation patterns in the LOTC, fusiform gyrus, parahippocampal gyrus, and posterior parietal cortex, consistent with the expected use of mid-level features in various visual tasks.

Additionally, we conducted searchlight RSA for each of the pose models with partial correlation controls over low-level HMAX C1 and mid-level SAM features. We chose not to regress out the semantic model and the joint-rotation based model in the partial correlation controls because they likely carry high-level semantics about poses. The results revealed distributed clusters similar to those observed in the standard RSA (*SI Appendix*, Fig. S9), suggesting that pose models indeed capture cortical sensitivity to poses rather than merely low-level or mid-level features.

## Discussion

In this study, we found distributed patterns of pose-related cortical responses across several brain regions, including early visual areas (V1, V2, V3) and category-selective areas (EBA, FBA, FFA), and parts of the parietal cortex. The distributed nature of cortical pose representations likely reflects both the complex computations required to extract body structure from images as well as different task objectives. Our results indicated cortical representations that encode intrinsic view-independent 3D pose in both LOTC and pSTS, and cortical representations that encode pose depth information in the LOTC.

### Computational Challenge of Estimating Pose in Natural Scenes.

Computational studies using deep convolutional neural networks (DCNNs) trained for specific recognition tasks have made significant progress in explaining how the visual system deals with image complexity (32, 33). One of the properties of deep convolutional networks is a monotonic increase in discrimination performance with layer depth, with some task-specific knowledge even at early layers (34). Parallel to this observation, as shown in Fig. 2, we indeed observed body-specific sensitivity as early as V1 to both the viewpoint and 2D/3D pose, after controlling low-level image and body features including HMAX C1. These body-related activations, though not accounted for by low-level controls, might be explained by features diagnostic for coarse-grained view and pose categorization, for example using the front vs. back of faces. Feature sharing is another property of deep networks, where the representation of common features for a broad range of recognition tasks is more similar in early layers but gradually becomes more selective with increased DCNN layer depth. Our research found pose clusters near the fusiform gyrus and LOTC, which are thought to be crucial for categorizing and understanding actions (35), as well as pose clusters in the posterior parietal cortex, involved in interpreting visual actions. It is important to note, however, that DCNNs exhibit notable differences in their approach to recognizing objects when compared to humans. In particular, DCNNs do not consistently classify objects based on the global shapes but seem to primarily depend on local features which functions in a manner similar to the ventral pathway (36). Recent evidence suggests that dorsal regions like IPS may compute the structural information that characterizes the global shape of an object (37, 38), which is consistent with our results for the pose clusters in the posterior parietal cortex.

We also found evidence of sensitivity to 3D pose in several regions including the LOTC and near pSTS. While these findings may have potential relevance to task requirements below, the representation of 3D structure may also be relevant to the computational challenges of image complexity. Despite the successes of deep convolutional networks in dealing with natural images, current pose estimation models fail to accurately estimate pose when a body is heavily occluded by other objects, surfaces, or other people (39). It is a conjecture that humans perform better at dealing with heavily occluded poses compared to current models, given that these occlusion cases are quite common in our everyday visual experience. Computational research has shown that strong prior knowledge of 3D depth body structure can provide more accurate pose estimation given occluded features than models that do not (2, 14, 40). Whether the LOTC or areas near pSTS are important for pose estimation given occlusion is an open question for future research.

### Diversity of Tasks May Drive the Emergence of Different Pose Representations.

While perceptual inferences based on 2D relationships between body parts can be sufficient for some tasks, knowledge of extrinsic depth relationships, in particular between the viewer and body parts has functional benefits for other tasks. For example, knowledge of the depths of another person’s limbs, such as arms and hands, is particularly important in personal interactions. As depicted in *SI Appendix*, Fig. S5, we found that seven out of eight subjects showed sensitivity to 3D view-dependent pose in the LOTC after removing dependency on 2D poses, suggesting that there may be cortical areas within the LOTC sensitive to the relative depths in poses. The lack of group-level significance could be due to the high correlation (0.97) between our 2D and 3D view-dependent pose models used in the partial correlation analysis, further compounded by the small number of subjects (n = 8) in the analysis and anatomical variations across individuals that are not fully compensated by the group normalization space. One future direction is to explore alternative pose model parameterizations to better distinguish 2D vs. 3D view-dependent posture information.

Estimations of the intrinsic 2D and 3D depth relationships between body parts can be useful for other task objectives. Some tasks, for example, involving decisions of action categories (e.g., distinguishing walking from running) can benefit from the computation of intrinsic pose structure, discounting viewpoint. Our previous psychophysical studies have shown that humans can discriminate 3D natural poses regardless of viewpoint changes, and that behavioral data are better explained by computational models using view-independent 3D pose information (41). In our study, distinct view-independent 3D pose clusters near the LOTC and pSTS were observed in group-level searchlight RSA results. Similar clusters were also found in the LOTC and the right pSTS using partial-correlation RSA. These areas may encode intrinsic 3D pose information independently of the viewpoint, as further suggested by the same result patterns from the joint rotation–based 3D view-independent pose control model despite its moderate correlation with the original view-independent 3D pose model (*SI Appendix*, Fig. S3). Past research has identified regions near the LOTC to be crucial for encoding body and body part information (3) as well as categorizing and understanding actions (35). The pSTS has been classically associated with several action-related visual tasks, including action observation (42), perception of human walking (43), action intention (44), and social interaction (45). The emergence of view-independent pose information in these regions aligns with the functional use of such intrinsic 3D pose information.

### Relationship to Past Studies of Body, Face (and Object) Representation in the Brain.

Studies on face and body patches in the macaque IT cortex showed greater tolerance to view changes and increased selectivity to face/body identity and posture in anterior patches (46–48). Similarly, we found a distributed cortical pose representation with increased view-tolerant selectivity for 3D human bodies from posterior to anterior regions, suggestive of underlying common characteristics between the processing principles for faces and bodies, even though bodies are more articulated than faces and ordinary objects.

FBA and EBA are two body selective regions identified by contrasting responses to simple body images with nonbody objects, suggesting their role in detecting the presence of a body and/or body parts. Recent studies further revealed posture encoding in the EBA with greater viewpoint tolerance arising anteriorly (8) as well as viewpoint-invariant decoding of body identity in the FBA (49). While these studies employed synthetic body stimuli with limited pose variations, our study develops body models that incorporate the geometric body structure over a wide range of poses while abstracting out other body-related properties, in particular retinotopic size and location. Aligned with these previous works, our findings also revealed 3D view-independent pose encoding in both EBA and FBA. However, we observe significant pose clusters mainly around EBA after controlling other view-dependent poses, suggestive that EBA may encode some intrinsic 3D pose information while FBA is less sensitive. This is supported by the positional overlap between our identified pose-sensitive clusters and body-related regions of interest, as well as higher neural correlations of pose models in EBA compared to other regions in our ROI-based RSA results, consistent with the causal role of EBA in detecting people from real-world scenes (50–52).

From the perspective of functional connectivity (FC), the EBA has been shown to interact with the FBA and occipitotemporal cortex (OTC), as well as superior temporal lobes, superior parietal lobes, and post- and precentral regions (53, 54). This aligns with the posited distributed cortical pose network across the LOTC, fusiform gyrus, posterior parietal cortex, and the temporal-parietal junction, suggesting the potential role of EBA in integrating and processing a 3D view independent pose configuration, subsequently contributing to planning and actions. In terms of encoding body retinotopic position and size, the higher neural correlations of the body position control models also suggest body positional encodings in EBA. Positional information has been found in some category-selective (face, body, scene, and object) regions (55–57). Thus, our results connect FBA and EBA to the processing of the spatial structure of the human body pose.

### Future Directions.

Moving forward, there are several intriguing avenues for further exploration of body pose representation and neural sensitivity. One open question pertains to how the brain handles self-occlusion when a limb is not visible. Our previous psychological studies on pose discrimination across different views (41) suggested that the visual system may infer the position and orientation of self-occluded joints. Regarding the mechanism for self-occlusion reasoning, one conjecture is that the visual system infers orientation of self-occluded joints from visible joints through relational representations. A previous fMRI study provided early evidence showing FBA sensitivity to aligned over misaligned body part relationships when viewed through apertures (58). Future directions would explore neural bases of structured integration of body parts and whether and where part connectedness is used in the body processing hierarchy.

Relatedly, in our experiment, all joints were presumed to contribute equally to the body geometric structure, given our focus on cortical sensitivity to the overall pose rather than body parts. Past work has identified topographic representations of individual body parts in the occipitotemporal cortex (5, 9). One future direction is to explore the cortical sensitivity to the geometric configuration for some subset of body parts (e.g., hand, wrist, elbow, and shoulder) by applying different weightings to these target joints.

Explaining variance in complex natural image responses with interpretable models as opposed to black box deep networks is in general a hard challenge. Despite statistical significance, our current modeling performance levels are still substantially below the noise ceiling. Our results provide positive evidence for cortical pose representations, but future work will need to integrate more pose dimensions (e.g., body position), construct comprehensive pose models and use techniques like voxel-wise fitting to address the performance gap.

The most important behavioral function of pose estimation is to interpret the action of a person. However, determining body pose by itself only partially constrains possible interpretations of an action. The visual system must also consider the spatial relationships between the body and other objects (also including head, surfaces, and other people). Further, while humans can infer an action based on a quick glance, motion information provides additional information about 3D body structure and the relationship of the body to other objects. The known close topographic relationship between hMT+ and classic body areas (59, 60) as well as the distributed pattern of activity found for biological motion (61) underscores the importance of understanding the relationship between pose, motion, and actions. It remains for future studies to discover how brain activation patterns for viewpoint and depth may differ between static and moving bodies, especially given the complexities of natural image input.

## Materials and Methods

### Stimulus Selection.

The Natural Scenes Dataset (15) contains 73,000 cropped color natural scene images from the MS COCO dataset (16). We aimed to select a subset of images that contain only single persons and cover a broad range of legitimate human body poses. To this aim, we used the ground truth person keypoint annotations provided by the MS COCO dataset. For each person in each image, the annotations consist of an enclosing person bounding box together with 2D image coordinates and visibility flags for 17 defined body keypoints, including five face keypoints (L/R eyes, nose, and L/R ears) as well as 12 limb keypoints (L/R shoulders, L/R elbows, L/R wrists, L/R hips, L/R knees, L/R ankles). We selected images with keypoint annotations for one and only one person inside the cropped image regions. As a next step, we further excluded single-person images under partial body presence, namely, where the persons were partially truncated by the image boundary. Specifically, we selected single-person images with 12 limb keypoints fully annotated. Face keypoints (eyes, nose, and ears) were not considered because these annotations were sometimes missing for persons with smaller areas in the images. Finally, we selected a subset of 4,450 images of full single persons under different poses.

### Natural Scenes Dataset.

The Natural Scenes Dataset consists of whole-brain, high-resolution (1.8-mm isotropic, 1.6-s sampling rate) 7T fMRI measurements of eight healthy adult subjects, while they each viewed thousands of color natural scenes over the course of 30 to 40 scan sessions. Each stimulus is presented between one to three times across multiple sessions for each subject, and each subject viewed a subset of the 4,450 natural pose stimuli. All stimuli in the NSD were adjusted to fill 8.4° of visual angle and were presented for 3 s. Subjects were trained to maintain central fixation, and the NSD data paper (15) shows evidence that this was largely successful (see extended data figure 4 in ref. 62).

### Pose Parameterization.

To parameterize natural poses, we extracted 3D pose information from complex natural scene images. MS COCO dataset does not provide ground truth 3D keypoint annotations or viewpoints. Therefore, we adopted an approach to use an off-the-shelf human 3D mesh reconstruction model to extract 3D pose information (17). Given a single RGB image in the wild, this model can reconstruct a full 3D human body mesh. The model was quantitatively evaluated on standard 3D joint estimation benchmarks and outperformed previous approaches that output 3D meshes (17). The viewpoint parameter for the 3D human body mesh is an axis-angle representation for the 3D body global rotation in SMPL format (13). The 3D rotation was transformed into a rotation matrix $R\in R^{3\times 3}$ for further processing. For body pose parameters, we transformed the 3D body mesh into a list of 3D joint locations with a trained joint location regressor from ref. 17. This joint list includes 19 joints (L/R ankles, L/R knees, L/R hips, L/R wrists, L/R elbows, L/R shoulders, neck, head, nose, L/R eyes, L/R ears). Thus, for each pose, we obtained a rotation matrix R for the body global rotation and a list of K=19 joint locations $p=[J_{1},J_{2},\ldots \ldots \ldots \ldots ,J_{K}]$, where $J_{k}\in R^{3}$. We did not perform additional normalization on these 3D joint coordinates because they were already in the same 3D body mesh reference frame.

To parameterize pose using 3D view-dependent joint locations $p_{3d\_v}=$$\left[J_{1}^{3d\_v},J_{2}^{3d\_v},\ldots \ldots \ldots \ldots ,J_{k}^{3d\_v}\right]$, we simply used these 3D joint coordinates $J_{k}^{3d\_v}=J_{k}\in R^{3}$.

To parameterize pose using 2D view-dependent joint locations $p_{2d}=[J_{1}^{\left(2d\right)},J_{2}^{\left(2d\right)},\ldots \ldots \ldots \ldots ,J_{K}^{\left(2d\right)}]$, we simply discarded the depth coordinate to make $J_{k}^{\left(2d\right)}\in R^{2}$.

To parameterize pose using 3D view-independent joint locations $p_{3d\_vi}=[J_{1}^{\left(3d\_vi\right)},J_{2}^{\left(3d\_vi\right)},\ldots \ldots \ldots \ldots ,J_{K}^{\left(3d\_vi\right)}]$, we reversed the global rotation to align poses to the same, original orientation

$$J_{k}^{\left(3d\_vi\right)}=R^{-1}J_{k},$$

where $R^{-1}$ is the inverse of the rotation matrix for the 3D global body rotation.

### Construction of Representational Dissimilarity Matrices (RDMs).

As aforementioned, we parameterized each natural pose into a pose vector, consisting of 2D view-dependent, 3D view-dependent, or 3D view-independent joint locations. Subsequently, we constructed each representational dissimilarity matrix for human poses by measuring the distance between pairs of body pose vectors. Specifically, to construct 3D view-independent aligned pose RDMs, we measured the dissimilarity between two aligned poses using Mean Per Joint Position Error which is used in much of the literature on 3D joint estimation. It measures the Euclidean distance averaged on all joints after aligning two poses. The dissimilarity between two poses $p_{i}^{\left(3d\_vi\right)}$ and $p_{j}^{\left(3d\_vi\right)}$ is measured as

$$d^{\left(3d\_vi\right)}\left(p_{i}^{\left(3d\_vi\right)},p_{j}^{\left(3d\_vi\right)}\right)=\frac{1}{K}\sum_{k=1}^{K}{∥J_{\mathrm{ik}}^{\left(3d\_vi\right)}-J_{\mathrm{jk}}^{\left(3d\_vi\right)}∥}_{2}.$$

Similarly, we can construct the 2D and 3D view-dependent pose RDM by measuring dissimilarity as

$$d^{\left(2d\right)}\left(p_{i}^{\left(2d\right)},p_{j}^{\left(2d\right)}\right)=\frac{1}{K}\sum_{k=1}^{K}{∥J_{\mathrm{ik}}^{\left(2d\right)}-J_{\mathrm{jk}}^{\left(2d\right)}∥}_{2},$$

$$d^{\left(3d\_v\right)}\left(p_{i}^{\left(3d\_v\right)},p_{j}^{\left(3d\_v\right)}\right)=\frac{1}{K}\sum_{k=1}^{K}{∥J_{\mathrm{ik}}^{\left(3d\_v\right)}-J_{\mathrm{jk}}^{\left(3d\_v\right)}∥}_{2}.$$

In order to construct the viewpoint RDM, we measured the viewpoint dissimilarity between pairs of bodies as the distance between the body global rotations in three-dimensions. We first transformed the associated 3D rotation matrix $R\in R^{3\times 3}$ into a unit quaternion q. Following ref. 63, we used the distance metric below to assess dissimilarity of two 3D body global rotations

$$d^{\left(v\right)}\left(q_{i},q_{j}\right)={\text{cos}}^{-1}\left|q_{i}\cdot q_{j}\right|.$$

To construct control RDMs, we obtained ground-truth person bounding boxes, person segmentations as well as image captions for each single-person image from COCO dataset annotations. For body position RDMs, we extracted the position of person bounding box centers in a polar coordinate system with origin at the center of the screen and calculated dissimilarity as differences in polar angles and polar distances. For the body size RDM, we obtained the relative size of the human body in each image using person segmentations and calculated body size differences as dissimilarity. To construct the semantic-based control RDM, we used Universal Sentence Encoder (64) to process image captions into normalized sentence embeddings. Dissimilarity between images was then characterized by the distance between their normalized sentence embeddings. For the HMAX model, we followed ref. 26 to extract C1 activations and calculated dissimilarity as the distances between the corresponding C1 activations. For the SAM mid-level RDM, we extracted the image embeddings from the SAM image encoder using the default ViT-H backbone. Dissimilarity values are computed as 1 – correlation between pairs of image embeddings.

To construct the joint rotation–based view-independent 3D pose control RDM, we first transformed the associated 3D joint rotations for K joints into a list of unit quaternion $Q_{i}=\left[q_{i1},q_{i2},\ldots \ldots \ldots \ldots ,q_{\mathrm{iK}}\right]$; then, we construct the control RDM by measuring dissimilarity as

$$d^{\left(3d\_vi\_\text{joint}\_\text{rotation}\right)}\left(Q_{i},Q_{j}\right)=\frac{1}{K}\sum_{k=1}^{K}{\text{cos}}^{-1}\left|q_{\mathrm{ik}}\cdot q_{\mathrm{jk}}\right|.$$

The dissimilarity is independent from viewpoint because $Q_{i}$ does not change under different viewpoint as long as the underlying 3D pose is the same.

All model RDMs are normalized before used in the representational similarity analysis. For each subject, model RDMs were normalized to the [0, 1] range by the following linear mapping.

$$x=\frac{x-\min(x)}{\text{max}\left(x\right)-\min(x)}.$$

### Searchlight-Based Representational Similarity Analysis.

The image-responses that we used in this paper are expressed as betas obtained from a general linear model (GLM) analysis. Here, we used GLM results provided by the NSD data release, with 1.8-mm volume resolution and version 2 of the GLM betas (betas_fithrf), in which the HRF is estimated for each voxel. We carried out the representational similarity analysis (RSA) using a searchlight approach in the individual volume space to investigate the relationship between the computed features and the brain activity. Spherical neighborhood of 100 voxels (approximately 5.2 mm in radius) was used for each searchlight. The multivariate analyses were performed using CosMoMVPA (65) and custom-written MATLAB functions (ver2017b, The MathWorks Inc.). The beta maps with the same images were first averaged, and then normalized across samples, so that each feature has zero mean and unit variance and contributes equally to the distance calculation. The neural RDM was derived using *1-correlation* as the distance metric. In the standard correlation analysis, we used four pose and viewpoint RDMs as target RDMs in our analysis. For each searchlight, the neural RDM was correlated with the normalized target RDM. Correlation values were assigned to the central voxel of each searchlight in each participant, which resulted in correlation maps for each model. In the partial correlation analysis, we adopted similar approaches but controlled for the effect of the other model RDMs before correlating the target RDM with the neural RDM. To get group-level results, the correlation maps in individual volume space were then resampled to the standard MNI space, using tools developed by NSD dataset authors (https://github.com/cvnlab/nsdcode). These tools are based on a nonlinear mapping from each subject’s T1 to the MNI152 T1 template as determined using FSL’s fnirt utility. Subsequently, correlation maps were Fisher z-transformed and entered in a group-level analysis to test the individual maps against zero using a one-tailed *t* test at each voxel. The t maps were then corrected with a cluster-based nonparametric Monte Carlo permutation test (cluster stat: max_sum, initial threshold *P* < 0.001; 10,000 iterations).

### ROI-Based Representational Similarity Analysis.

In ROI-based analysis, we used ROIs defined in the NSD dataset with a specific focus on V1-V4 and face- and body-selective regions. Specifically, V1, V2, and V3, and area hV4 were defined based on results of a population receptive field (pRF) experiment. Category-selective ROIs, including extrastriate body area (EBA) and fusiform face area (FFA), etc., were defined based on results of the functional localizer (fLoc) experiment developed by the Grill-Spector lab (66). See *SI Appendix*, Fig. S10 for ROI visualizations. We selected voxels with t-values greater than 8.0 for EBA, while we used all associated voxels for the other ROIs in the analysis. The representation similarity analysis was done on a subject-by-subject basis. For each subject, we extracted selected voxel responses to single-person images used in searchlight analysis and built neural RDMs as *1-correlation* between different trials. We subsequently calculated the Pearson correlation between neural RDMs and target model RDMs for each ROI. In the partial correlation analysis, we controlled for the effect of control model RDMs before correlating the target model RDM with the neural RDM. Finally, we reported the correlation coefficient in each ROI from each individual subject along with the mean and SD of the correlation across all eight subjects.

We followed the method developed in ref. 23 to obtain single-subject RSA noise ceilings, referred to as within-subject representational similarity matrices (RSMs) where noise accounts for trial-to-trial variability in a subject. Given that 38.8% of stimuli were presented at most twice in four out of eight subjects, we measured the noise ceiling with two trials to cover most of the stimuli. Within each brain ROI, we estimated multivariate Gaussian distributions for signal and noise, assuming that observed responses result from sum of samples from these distributions. Subsequently, we adjusted the signal distribution post hoc to ensure that it aligns with the empirically observed reliability of RSMs across independent data splits. Noise ceilings were derived through Monte Carlo simulations, wherein a noiseless RSM (generated from the estimated signal distribution) was correlated with RSMs constructed from noisy measurements (generated from both the signal and noise distributions). The mean noise ceiling and SEM across subjects are depicted in Fig. 5.

## Supplementary Material

Appendix 01 (PDF)

## Data Availability

fMRI data and analysis code data have been deposited in NSD_Body_Pose (Raw fMRI data we used is available from https://naturalscenesdataset.org (67). Image data we used is available from https://cocodataset.org/ (68). Processed fMRI data and custom code will be available at Open Science Framework (DOI: 10.17605/OSF.IO/N3ADH) (69).
