# Supplementary material for: Natural scenes reveal diverse representations of 2D and 3D body pose in the human brain
Source: Proc Natl Acad Sci U S A. 2024 Jun 3;121(24):e2317707121. doi: 10.1073/pnas.2317707121 (PMC11181088; doi:10.1073/pnas.2317707121)
Supplement: Supplementary file 1 — Appendix 01 (PDF) [file pnas.2317707121.sapp.pdf]

PNAS

Supporting Information for
Natural scenes reveal diverse representations of 2D and 3D body pose in the human brain

Hongru Zhu, Yijun Ge, Alexander Bratch, Alan Yuille, Kendrick Kay, Daniel Kersten

Yijun Ge
Email: yijun.ge@riken.jp

This PDF file includes:

Figures S1 to S10
SI References

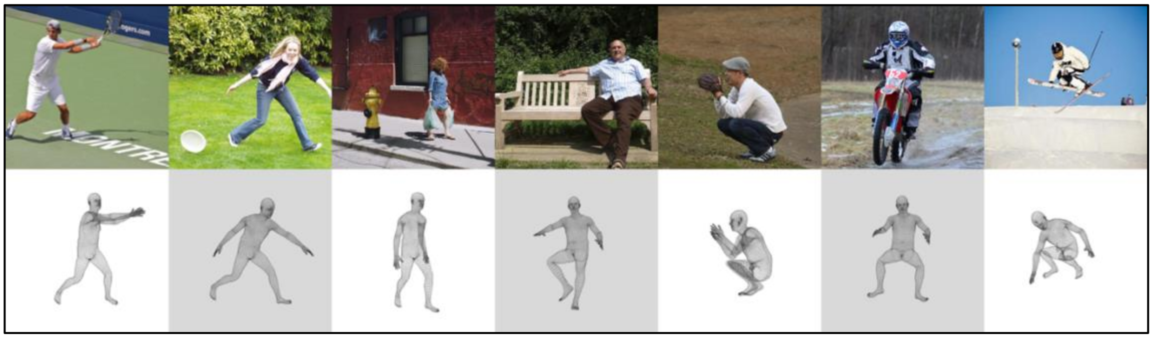

Figure S1. Example natural images of single person used in Natural Scenes Dataset (first row), together with corresponding reconstructed 3D meshes (second row) capturing the person's pose.

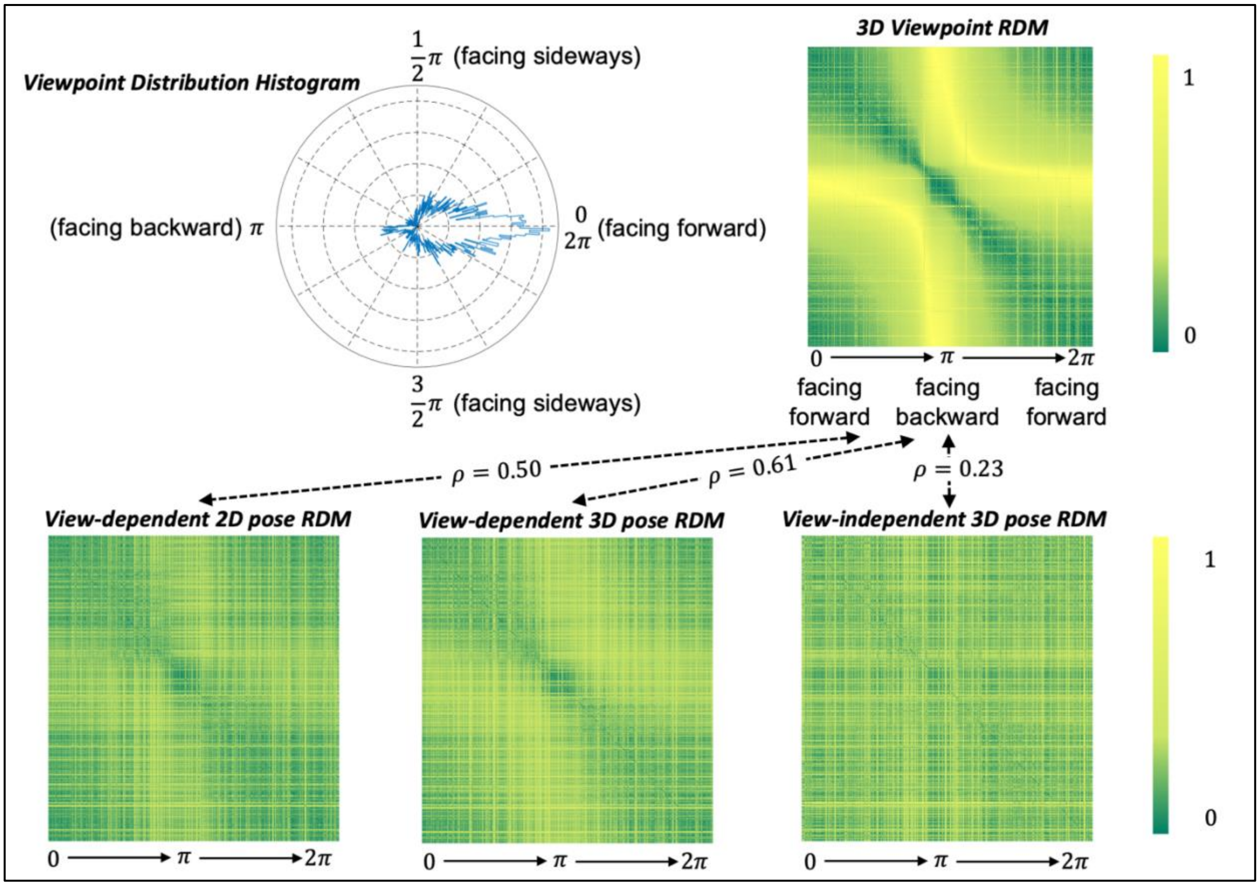

Figure S2. Viewpoint distribution for 4,450 natural pose images and RDMs with images ordered by their viewpoint angle ranging from $\mathbf{0}$ to $\mathbf{2\pi}$. A viewpoint of $\mathbf{0}$ or $\mathbf{2\pi}$ corresponds to forward-facing bodies in the images while viewpoint $\boldsymbol{\pi}$ corresponds to backward-facing bodies. Correlation between RDMs were noted as the Pearson correlation coefficient along the dashed lines connecting different RDMs. View-dependent 2D and 3D pose RDMs have similar patterns and higher correlation with the viewpoint RDM compared with the view-independent 3D pose RDM.

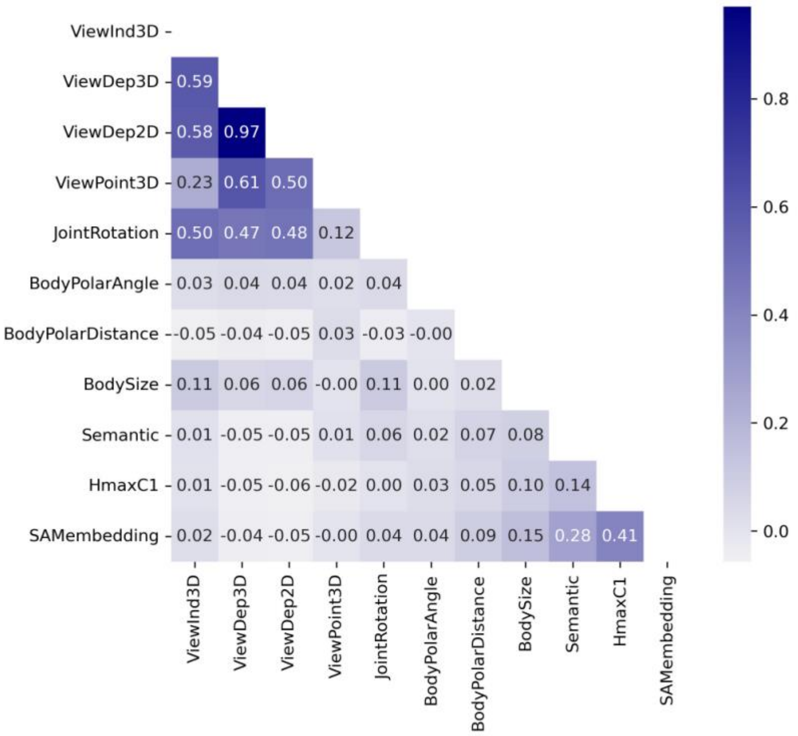

Figure S3. Pearson correlation between different target pose and control RDMs. Correlations between pose models are higher while correlation across pose and control models are relatively small.

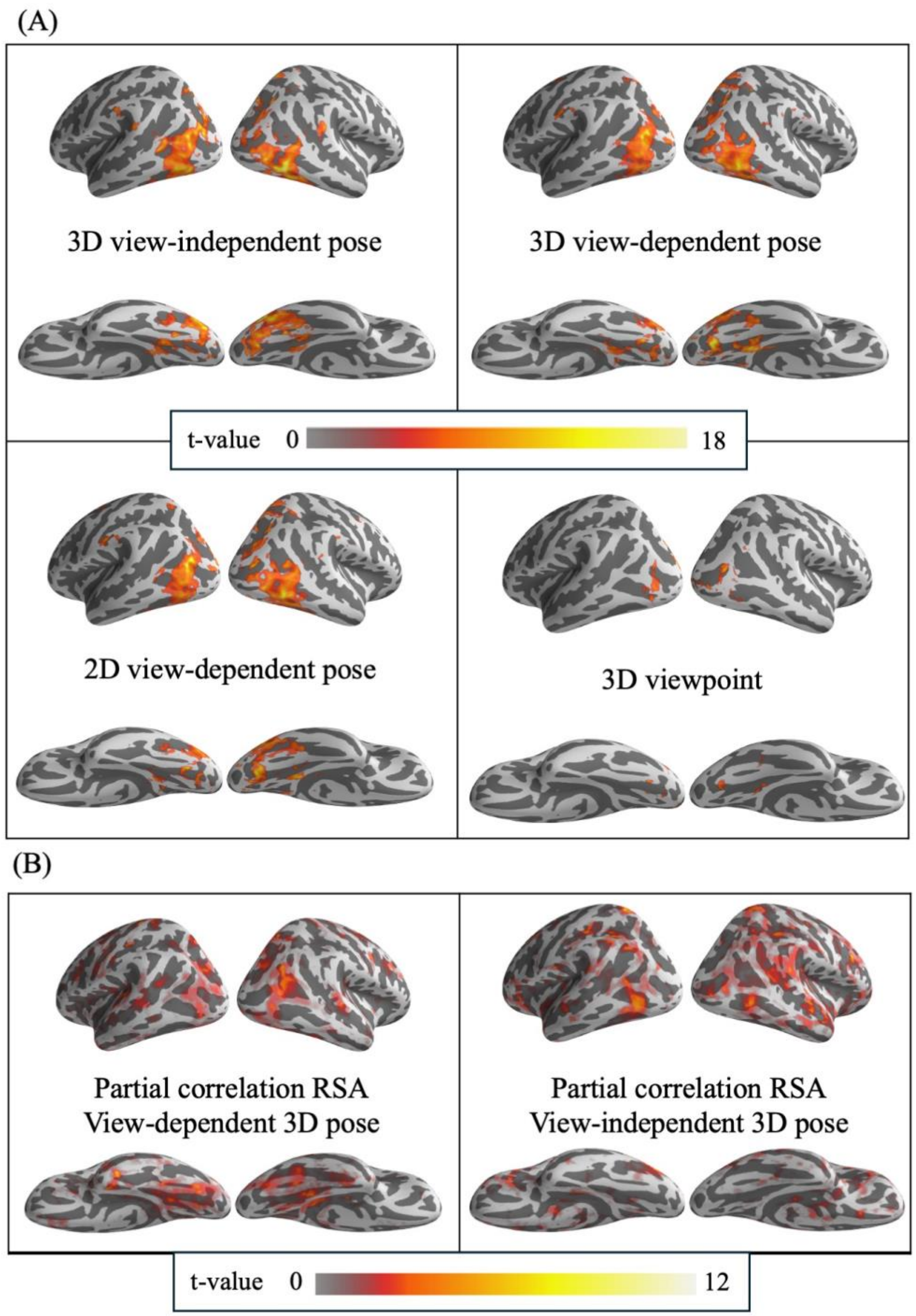

Figure S4. Inflated brain views for group level results. (A) Inflated view of standard RSA t-value maps for statistically significant clusters as shown in Figure 2. (B) Left: Inflated view of partial correlation RSA t-value map for the view-dependent 3D pose model as shown in Figure S5. Right: Inflated view of partial correlation RSA t-value map for the view-independent 3D pose model as shown in Figure 3.

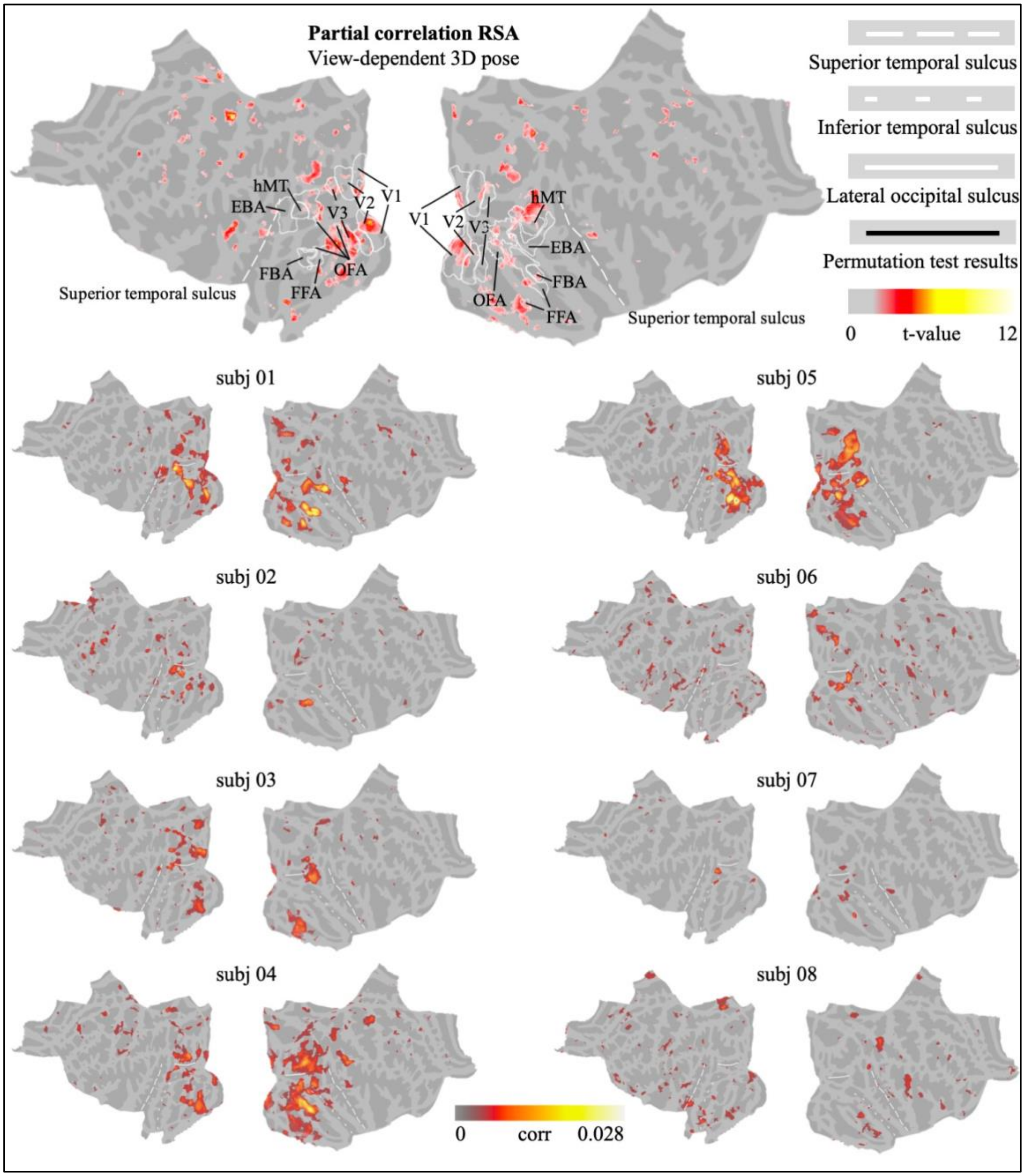

Figure S5. Group-level results (top) as well as individual partial correlation maps highlighting areas responsive to relative depth in poses, obtained after regressing out 2D view-dependent components from the 3D view-dependent pose model. Color maps show t-values and partial correlation values respectively for group-level and individual plots. None of the clusters from group-level results passed the cluster-based permutation test (cluster stat: max sum; init p<0.001). However, seven out of eight subjects (except for subj 08) showed clusters in LOTC with higher partial correlation values after removing dependency on 2D pose. Inflated surface maps of the individual results are included in Figure S6. Inflated surface maps of the group-level results are included in Figure S4-(B).

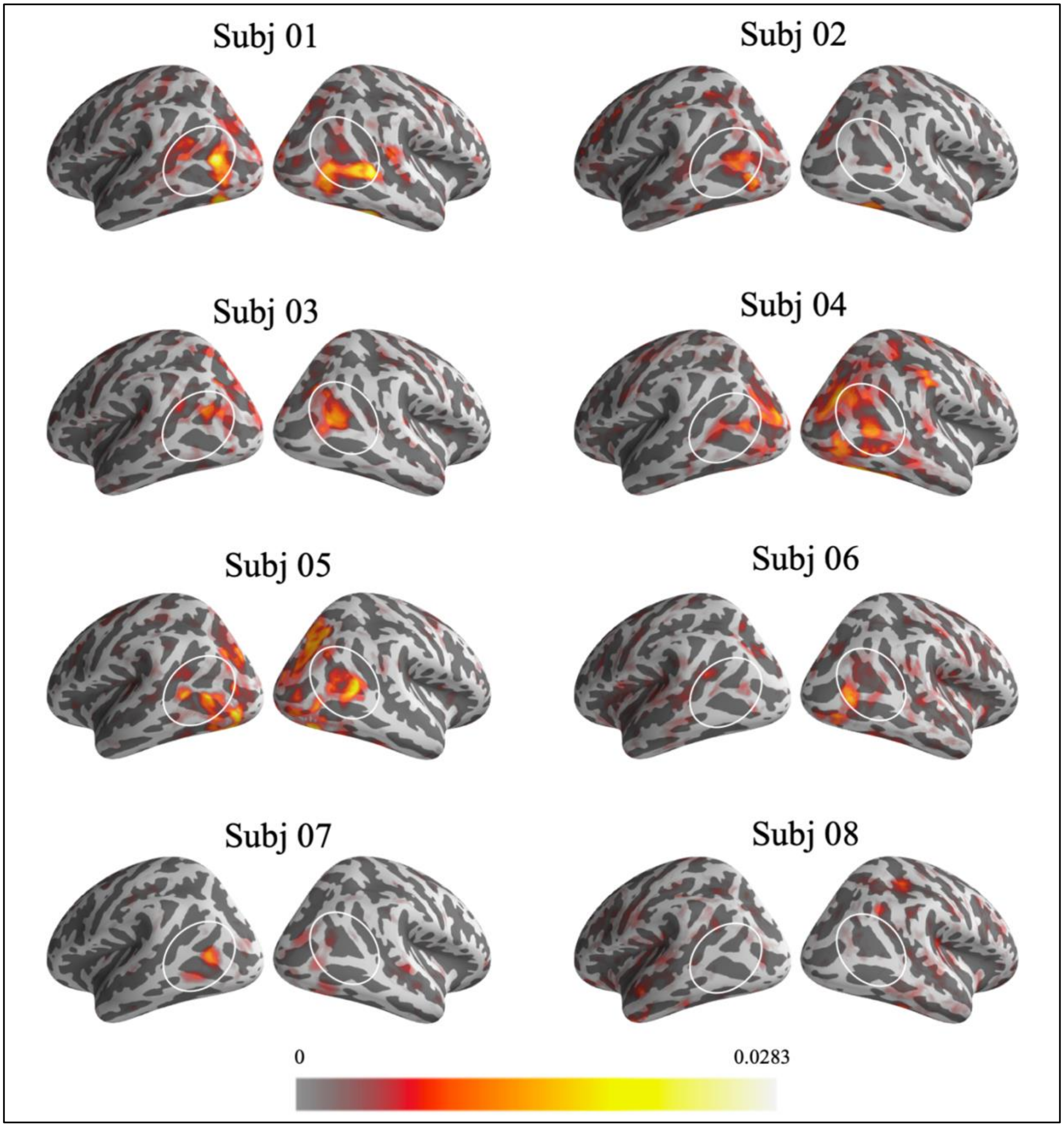

Figure S6. Individual partial correlation maps highlighting areas responsive to relative depth in poses. Color maps show the partial correlation values from the searchlight-based RSA for view-dependent 3D pose model while regressing out view-dependent 2D pose model. White circles indicate the approximate location of human LOTC defined as previously described in (1).

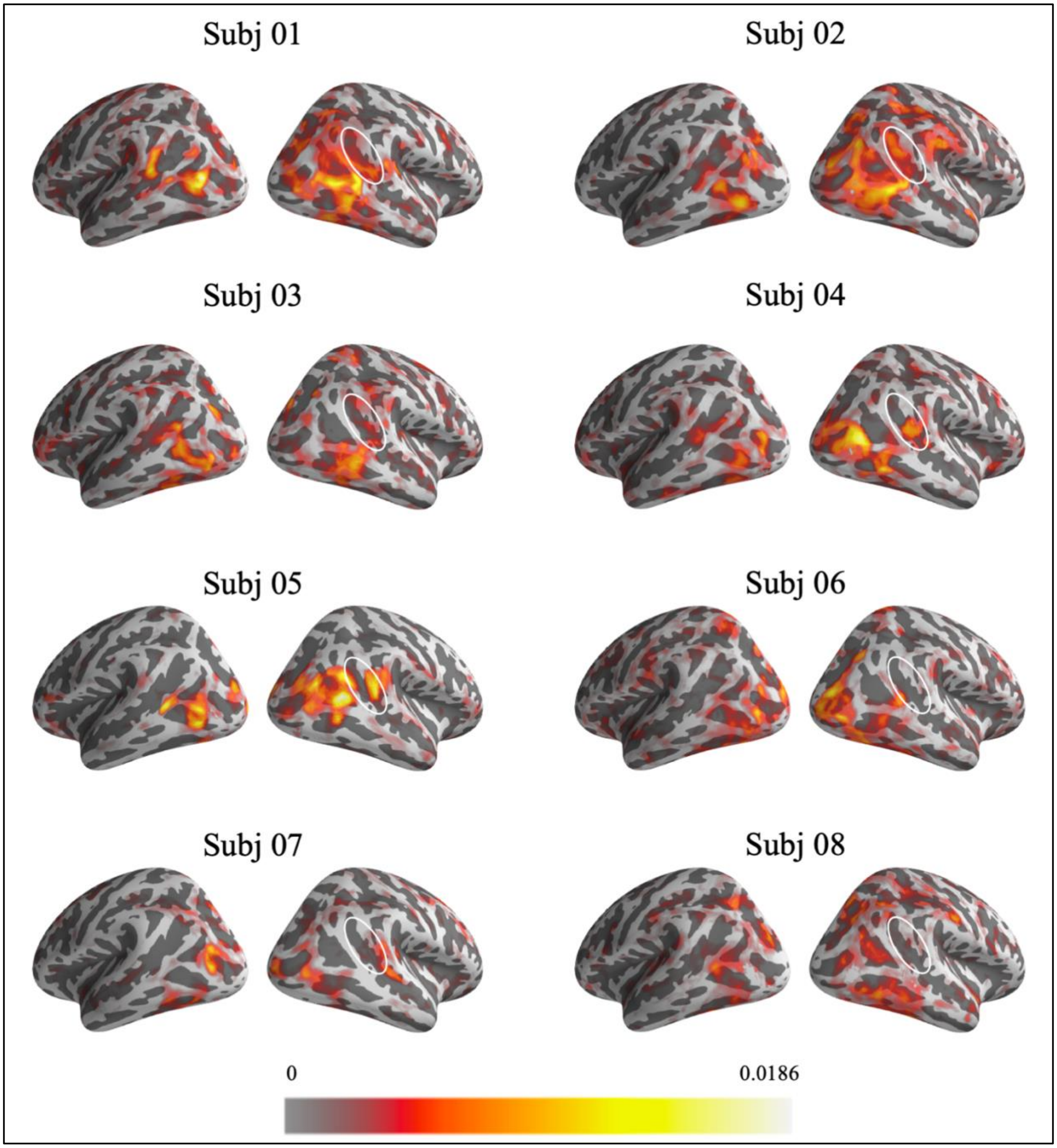

Figure S7. Individual partial correlation maps highlighting areas responsive to 3D intrinsic, view-independent pose information. Color maps show the partial correlation values from the searchlight-based RSA for view-independent 3D pose model while regressing out the effect from view-dependent 3D pose model, view-dependent 2D pose model and viewpoint RDM. White circles indicate the approximate location of right pSTS.

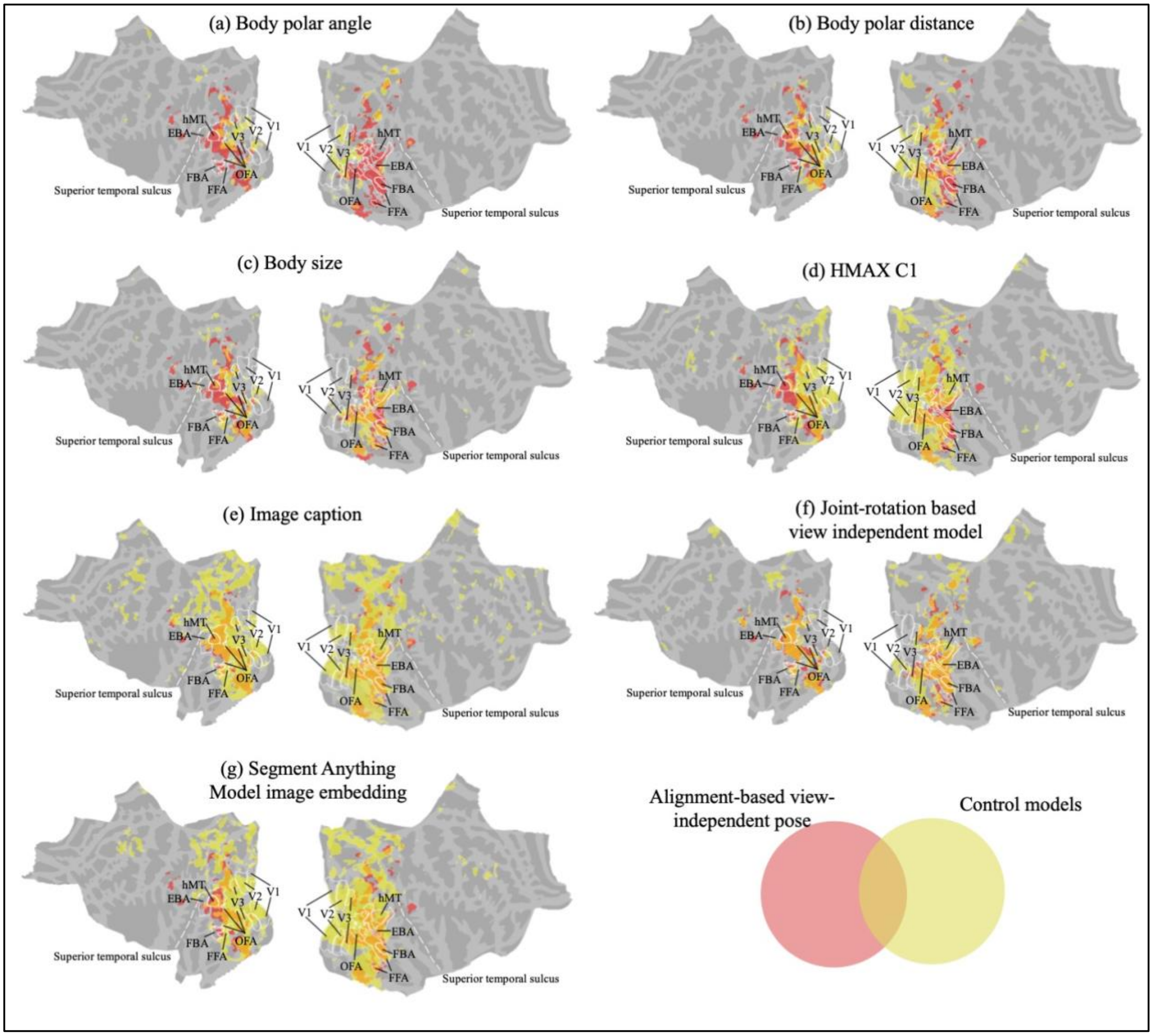

Figure S8. Standard searchlight-based RSA results for the control models (marked by yellow), including (a-c) three control models for body positions and sizes, (d) a low-level HMAX C1 model, (e) a higher-level semantic model based on image captions, (f) a joint rotation-based view-independent pose model and (g) a mid-level feature model based on Segment Anything Model image embeddings. Red regions depict the RSA results from 3D view-independent pose model as a comparison. Searchlight-based RSA procedures for control models are the same as those previously used for evaluating pose models.

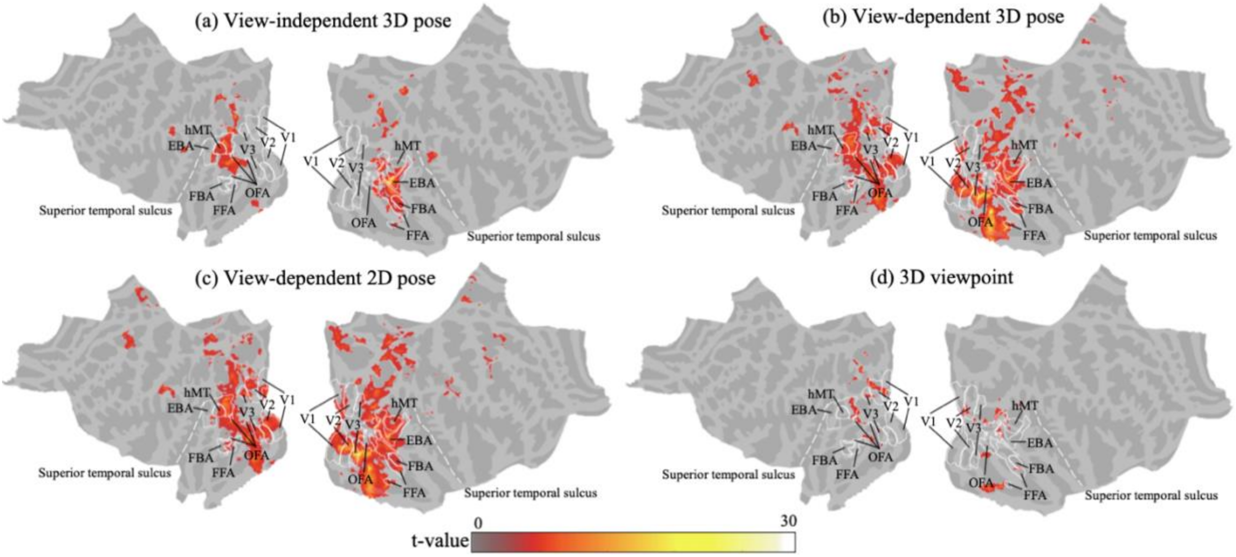

Figure S9. Group-level searchlight-based RSA results for pose model after regressing out low-level HMAX C1 and mid-level SAM features. Color maps show t values for significant clusters that passed a cluster-based nonparametric analysis with Monte Carlo permutation (cluster stat: max sum; initial $p<0.001$). The correlation maps for each participant were first Fisher transformed to normal distribution and then the t scores were measured. All pose models showed clusters in LOTC. View-independent 3D pose results also showed unique clusters along the right posterior STS (marked by dashed white line).

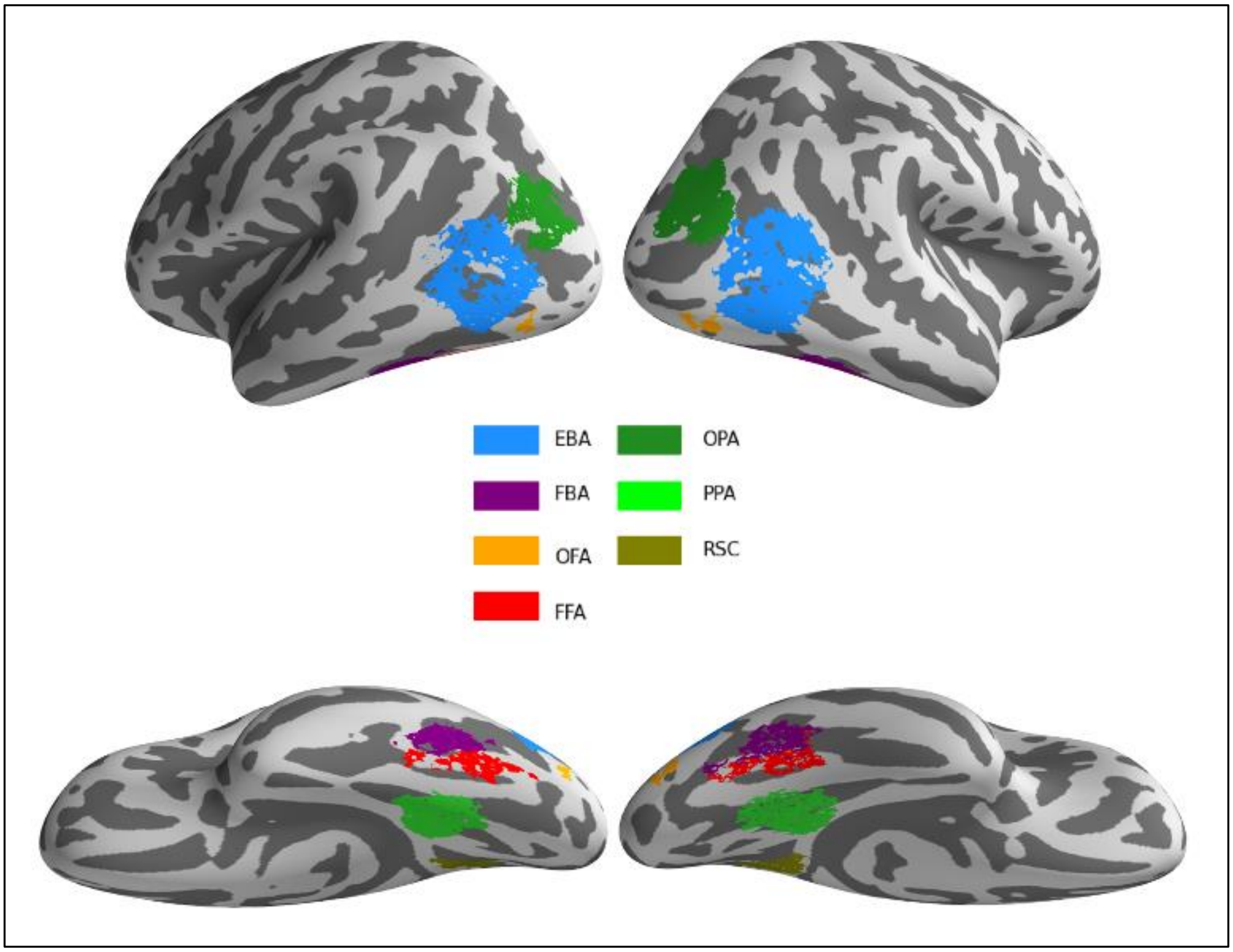

Figure S10. Group-level functional localizer results for body, face and place selective areas across all the subjects. Color map threshold is 62.5% (five out of eight subjects). The body selective areas include EBA and FBA. The face selective areas include FFA and OFA. The place selective areas include PPA, OPA and RSC.

SI References

1. A Lingnau, PE Downing, The lateral occipitotemporal cortex in action. *Trends in cognitive sciences* **19**, 268–277 (2015).
